# Sugammadex versus anticholinesterase reversal of neuromuscular blockade in adult surgical patients: a systematic review and meta-analysis

**DOI:** 10.3389/fmed.2026.1891778

**Published:** 2026-10-07

**Authors:** Faris Suleiman Saleh Aldobekhi, Mohammed Fahad Aldhahi, Fahad Awadh Almalki, Mohammed Aljahdali, Hassan Alshamrani, Somaia Sameer Akbar, Sultan Awwadh Almalki

**Affiliations:** 1 Department of Surgical Subspecialities, College of Medicine, Majmaah University, Al Majmaah, Saudi Arabia; 2 King Salman Armed Forces Hospital, Tabuk, Saudi Arabia; 3 Department of Anesthesia, King Abdulaziz Medical City, Ministry of the National Guard Health Affairs, Riyadh, Saudi Arabia; 4 SEHA Virtual Hospital, Makkah, Saudi Arabia; 5 King Abdulaziz Medical City, Riyadh, Saudi Arabia

**Keywords:** anticholinesterase agents, meta-analysis, neuromuscular blockade reversal, postoperative outcomes, sugammadex

## Abstract

**Background:**

Residual neuromuscular blockade after general anaesthesia is a cause of postoperative pulmonary complications and delayed recovery. Sugammadex, a relaxant-binding agent, is a fast-acting antidote for aminosteroid neuromuscular block which works through encapsulation, in contrast to acetylcholinesterase inhibitors like neostigmine. This study evaluated the effectiveness, postoperative recovery and complications associated with sugammadex versus conventional anticholinesterase reversal drugs in adult surgical patients.

**Method:**

This systematic review and meta-analysis was done based on the PRISMA 2020 statement. Databases like PubMed/MEDLINE and Scopus were used to search the literature from January 2000 to December 2025. Studies comparing the use of sugammadex to anticholinesterase agents in adult surgical patients were included, both randomised and non-randomised. Two independent reviewers screened, extracted data and assessed the risk of bias. Analyses were carried out in RevMan 5.3 software using random-effect models; the odds ratio (OR) for dichotomous outcomes and the mean difference (MD) for continuous outcomes were used with 95% confidence intervals (CI). Evidence certainty was assessed using the GRADE approach.

**Results:**

Total of ten studies (*n* = 4,082) were included; 5 randomised control trials and 5 observational studies. There was no statistically significant reduction in postoperative pulmonary complications with sugammadex compared to anticholinesterase (3 studies, *n* = 688; OR 0.75, 95% CI 0.47–1.20; *p* = 0.23; I^2^ = 77%). There was faster recovery of neuromuscular function with sugammadex, however, there was very high heterogeneity among the studies and the pooled estimate cannot be interpreted as one single effect size (2 studies; I^2^ = 100%). Recovery time after surgery was reduced (3 studies, *n* = 260; MD -6.66 min, 95% CI −6.96 to −6.35; *p* < 0.00001; I^2^ = 87%) and time to first bowel movement was also earlier (2 studies, *n* = 559; MD −13.81 h, 95% CI -19.16 to −8.45; *p* < 0.00001; I^2^ = 0%). There were no statistically significant differences for PACU stay (MD 1.55 min, 95% CI −1.60 to 4.69), readmission rate (OR 0.93, 95% CI 0.62–1.39) and postoperative nausea/vomiting (OR 0.86, 95% CI 0.48–1.56).

**Conclusion:**

Faster neuromuscular recovery, shorter recovery time after surgery and early return of bowel function was associated with the use of sugammadex rather than the anticholinesterase agents. There were no differences found for postoperative pulmonary complications, PACU stay, readmission rate or nausea and vomiting. These results are based on small number of clinically heterogeneous studies, in which only 2–4 studies were used in pooling and thus should be considered as preliminary results.

**Systematic review registered:**

CRD420261364292.

## Introduction

Recently neuromuscular blocking agents (NMBAs) have been extensively employed to achieve muscle relaxation and improved operating conditions, thus reducing the incidence of adverse events due to the postoperative condition (1). Residual neuromuscular blockade with muscle weakness is known to be among the contributing factors to postoperative pulmonary complications (PPCs) and the PACU delayed recovery (2). Acetylcholinesterase inhibitors such as neostigmine and pyridostigmine are traditionally used to antagonize residual neuromuscular blockade. However, since they have muscarinic properties, they are combined with an anticholinergic drug such as atropine or glycopyrrolate (3). Sugammadex is the modified form of *γ*-cyclodextrin, which forms a 1:1 complex with aminosteroidal neuromuscular blockers, including rocuronium or vecuronium. Thus, the complex reduces the free plasma concentration of the drug, and blocks the blockade, but does not affect the neuromuscular junction (4). A Cochrane review of 41 studies demonstrated that sugammadex reversed rocuronium-induced neuromuscular blockade faster than neostigmine and resulted in approximately 40% fewer adverse events (5). Sugammadex captures rocuronium and vecuronium, causing their dissociation from nicotinic acetylcholine receptors. The formed complex is pharmacologically inactive and is eliminated through kidneys. No cardiovascular or haemodynamic adverse effects have been identified in the results of preclinical and clinical trials (6). The effects of anticholinesterase agents include the motor end terminal stimulation, which causes the increased secretion of acetylcholine (Ach), resulting in the elimination of muscle relaxants from receptors. This leads to the reversible inactivation of ACh esterase enzyme. In addition to the blockage mechanism, there are several muscarinic effects caused by cholinesterase inhibitors in the cardiovascular, gastrointestinal and pulmonary systems. Despite the attenuation of these effects by atropine sulfate or glycopyrrolate, the use of these drugs introduces additional anticholinergic load (7). Paralysis recurrences have been reported during using sugammadex, especially among patients with cardiac surgery and when using fixed dose regulation despite the quantitative neuromuscular evaluation. In addition to the advantages of sugammadex, its drawbacks are cost-effectiveness and the management of certain patient conditions such as renal failure or obesity. While the traditional anticholinesterase reversal agents such as neostigmine have limitations including unpredictability of the effect, lengthy period of reversal and parasympathetic effects (8). Neostigmine administered at a shallow depth of blockade can even cause muscle weakness (7, 9). Sugammadex dosage depends on the body weight and is titrated according to the depth of the blockade with 2 mg/kg being prescribed at TOF second twitch reappearance, 4 mg/kg at post-tetanic count 1–2 and 16 mg/kg in case of rapid reversal after rocuronium 1.2 mg/kg administration (9). Sugammadex promotes the full reversal of the deep neuromuscular blockade within 2 to 4 min, significantly reducing the risk of residual paralysis, although it is still associated with possible respiratory complications such as bronchospasm (4, 8). The aims and objectives of the study are to evaluate and compare the safety and efficacy of sugammadex and acetylcholinesterase inhibitors for the reversal of the neuromuscular blocking agents. Besides, the study evaluated the duration of recovery, the occurrence of postoperative pulmonary complications and several other clinical outcomes in the post-anaesthesia care unit (PACU). Although there have been numerous meta-analyses devoted to this topic, most of them are limited to randomised trials or single outcome domains, while few examined recovery, gastrointestinal and resource utilization outcomes together in randomised and observational studies. The current review was performed to address this gap.

## Method

### Research question and design

The review was performed and reported as per the Preferred Reporting Items for Systematic Reviews and Meta-Analyses (PRISMA) 2020 reporting guidelines (10). The review protocol was not registered in advance in PROSPERO database, which is considered as a limitation, but the eligibility criteria, outcomes and methods of analysis were stated before the data collection and no changes were made afterwards. Reporting is done using the PECOS criteria: Population - adults undergoing surgery under general anesthesia and requiring neuromuscular blockade; Exposure - sugammadex; Comparator – neostigmine and/or pyridostigmine, with or without anticholinergic; Outcome measures - postoperative pulmonary complications, time of neuromuscular recovery, postoperative recovery time, time of first defecation, PACU length of stay, hospital admission rate and postoperative nausea and vomiting; Designs – randomised controlled trials and comparative observational studies.

### Eligibility criteria and group definition

#### Inclusion criteria

- Studies evaluating adult patients undergoing surgical procedures requiring neuromuscular blockade under general anaesthesia.
- Studies comparing Sugammadex with conventional anticholinesterase agents such as neostigmine or pyridostigmine, with or without anticholinergic drugs.
- Randomised controlled trials, prospective studies, retrospective cohort studies, and observational studies reporting comparative outcomes.
- Studies reporting at least one relevant perioperative outcome such as pulmonary complications, neuromuscular recovery time, postoperative recovery time, gastrointestinal recovery, PACU stay, readmission rate, or postoperative nausea and vomiting.
- Studies involving adult surgical populations across different surgical specialties including colorectal, bariatric, thoracic, laparoscopic, or spinal procedures.
- Studies providing sufficient quantitative data for extraction and meta-analysis.
- Articles published in peer-reviewed journals and available in full-text form.

#### Exclusion criteria

- Studies conducted exclusively in paediatric populations.
- Studies without a comparison group between Sugammadex and anticholinesterase reversal agents.
- Case reports, case series, editorials, narrative reviews, conference abstracts, and letters without original data.
- Studies lacking sufficient quantitative outcome data required for statistical synthesis.
- Duplicate publications or secondary analyses of the same dataset where primary data were already included.
- Experimental or animal studies not involving human surgical patients.

### Literature search

PubMed/MEDLINE and Scopus were searched from 1 January 2000 to 31 December 2025. Search strategy consisted of using controlled vocabulary as well as free-text terms for the intervention and comparator along with the surgical population, as follows: (“sugammadex” OR “Bridion”) AND (“neostigmine” OR “pyridostigmine” OR “anticholinesterase” OR “acetylcholinesterase inhibitor” OR “neuromuscular reversal”) AND (“surgery” OR “colorectal” OR “bariatric” OR “laparoscopic” OR “thoracic” OR “spine”). No language restriction was applied. Hand searching of reference lists from retrieved papers as well as systematic reviews was performed to identify additional eligible trials. The search was limited to the peer-reviewed literature only and no grey literature (trial registries, conference proceedings) was included, which is recognised as a limitation. Searches results were downloaded into reference manager and duplicates removed. Screening of title/abstract followed by full-text screening were done by two reviewers independently, with disagreements resolved by discussion or by a third reviewer.

### Data extraction and outcome measures

Data extraction was performed independently by two reviewers who used a predefined data extraction form that was created prior to the analysis process. Information about the selected articles was systematically extracted by two reviewers to maintain consistency and avoid any possible errors in the process. The variables to be extracted included the first author’s surname, the year of publication, the location where the study took place, the study design used, the number of participants in the study, the characteristics of the study population, the type of surgery performed, the agents used for reversal of the neuromuscular blockade, and the outcome of interest reported in the study. In case of any disagreement between reviewers in the data extraction process, the problem was solved through discussion. Outcome measure in the analysis of the results was related to the perioperative outcomes that were reported in the studies selected. Some of the outcomes included post-operative pulmonary complications, recovery of neuromuscular, time to recovery from the surgery, time to first bowel movement, PACU stay time, readmission rate, and post-operative nausea and vomiting. The outcomes such as postoperative pulmonary complications, readmission, and postoperative nausea and vomiting were estimated using odds ratio with 95% confidence interval while the continuous outcomes such as recovery time and gastrointestinal recovery were estimated using mean differences with their confidence intervals.

### Quality assessment

Risk of bias was assessed independently by two reviewers using design-appropriate instruments. Randomised controlled trials were appraised with the Cochrane Risk of Bias 2 (RoB 2) tool across the domains of randomisation process, deviations from intended interventions, missing outcome data, measurement of the outcome and selection of the reported result, with each trial rated as low risk, some concerns, or high risk. Non-randomised studies were appraised with the ROBINS-I tool across its seven domains spanning the pre-intervention stage (confounding, selection of participants), the intervention stage (classification of interventions) and the post-intervention stage (deviations from intended interventions, missing data, measurement of outcomes, selection of the reported result), with each study rated as low, moderate, serious or critical risk. Because the two instruments are not interchangeable, ratings are reported separately by design in Table 1 and are not combined into a single summary score. Discrepancies were resolved by consensus. The certainty of evidence for each pooled outcome was rated using the GRADE approach as high, moderate, low or very low, with observational evidence starting at low certainty and rated down for risk of bias, inconsistency, indirectness, imprecision and suspected publication bias (Table 2).

**Table 1:** Risk of bias in the randomised controlled trials, assessed with the cochrane risk of bias 2 (RoB 2) tool.

Study (design)	D1 Randomisation process	D2 Deviations from intended interventions	D3 Missing outcome data	D4 Measurement of the outcome	D5 Selection of the reported result	Overall risk of bias
Ajetunmobi 2025 (11)	Low	Low	Low	Low	Low	Low
Han 2021 (12)	Low	Some concerns	Low	Some concerns	Some concerns	Some concerns
Ledowski 2021 (13)	Low	Low	Low	Low	Low	Low
Mraovic 2021 (14)	Low	Some concerns	Some concerns	Low	Low	Some concerns
Yu 2022 (15)	Low	Low	Some concerns	Some concerns	Low	Some concerns

**Table 2:** Risk of bias in the non-randomised studies, assessed with the ROBINS-I tool.

Study (design)	D1 confounding	D2 Selection of participants	D3 Classification of interventions	D4 Deviations from intended interventions	D5 Missing data	D6 Measurement of outcomes	D7 Selection of the reported result	Overall risk of bias
Chae 2019 (16)	Moderate	Moderate	Low	Low	Low	Low	Moderate	Moderate
Hunt 2020 (17)	Serious	Moderate	Low	Moderate	Moderate	Moderate	Moderate	Serious
Oh 2021 (18)	Moderate	Moderate	Moderate	Moderate	Low	Moderate	Moderate	Moderate
Serrano 2021 (19)	Moderate	Low	Low	Moderate	Low	Low	Moderate	Moderate
Traeger 2022 (20)	Serious	Moderate	Low	Moderate	Moderate	Moderate	Moderate	Serious

### Statistical analyses

Analyses were performed in RevMan 5.3 (The Nordic Cochrane Centre, The Cochrane Collaboration, 2014). Pairwise meta-analyses were undertaken for each outcome and summary estimates reported with 95% confidence intervals. Dichotomous outcomes were summarised as odds ratios using the Mantel–Haenszel method. Continuous outcomes were summarised as mean differences, since all contributing studies reported the same outcome on the same scale (minutes or hours); standardised mean differences were therefore not required. Where studies reported medians with interquartile ranges, means and standard deviations were estimated using established methods for conversion of summary statistics. Heterogeneity was quantified using the Cochran Q test and the I^2^ statistic and interpreted as low (0–40%), moderate (30–60%), substantial (50–90%), or considerable (75–100%). Because the included studies differed in design, surgical population, depth of blockade, reversal dosing and outcome definition, a common treatment effect could not be assumed, and DerSimonian–Laird random-effects models were applied to all pooled analyses; the τ^2^ and 95% prediction interval are reported alongside each estimate where three or more studies contributed. Fixed-effect estimates are presented in the forest plots as generated by RevMan and are retained for transparency, but the random-effects estimate is the primary result for every outcome. Sensitivity analysis by leave-one-out omission was performed for outcomes in which a single study contributed more than 50% of the weight. Assessment of publication bias by funnel plot asymmetry and by Egger regression was not undertaken because fewer than ten studies contributed to any single outcome, below the threshold at which these tests have adequate power to distinguish asymmetry from chance.

## Results

The PRISMA flow diagram illustrates the systematic process used for identification, screening, eligibility assessment, and final inclusion of studies in the review. Initially, a total of 99 studies were identified through database searches. Prior to screening, 11 duplicate records were removed, and an additional 4 studies were excluded because they were not relevant to the research topic. After these removals, 84 studies remained for the screening stage. During the screening process, titles and abstracts were examined, resulting in the exclusion of 23 studies that did not report relevant outcomes related to neuromuscular blockade reversal. Consequently, 61 studies were sought for full-text retrieval. Among these, 38 studies were excluded because they represented other study designs, lacked sufficient data for analysis, or were experimental studies not meeting the eligibility criteria. Of the 23 reports sought for retrieval at this stage, 13 could not be obtained in full-text form and were recorded as reports not retrieved, leaving 10 reports assessed for eligibility. It is acknowledged that the inability to retrieve 13 of 23 reports (57%) is a substantial shortfall that may have introduced selection bias, and this is addressed in the limitations. After applying all eligibility criteria, a total of 10 studies met the inclusion criteria and were incorporated into the qualitative and quantitative synthesis of the systematic review and meta-analysis. The 10 included studies comprised five randomised controlled trials (11–15) and five comparative observational studies (16–20), contributing 4,082 patients in total. No study was excluded on grounds of language (Figure 1).

**Figure 1:**
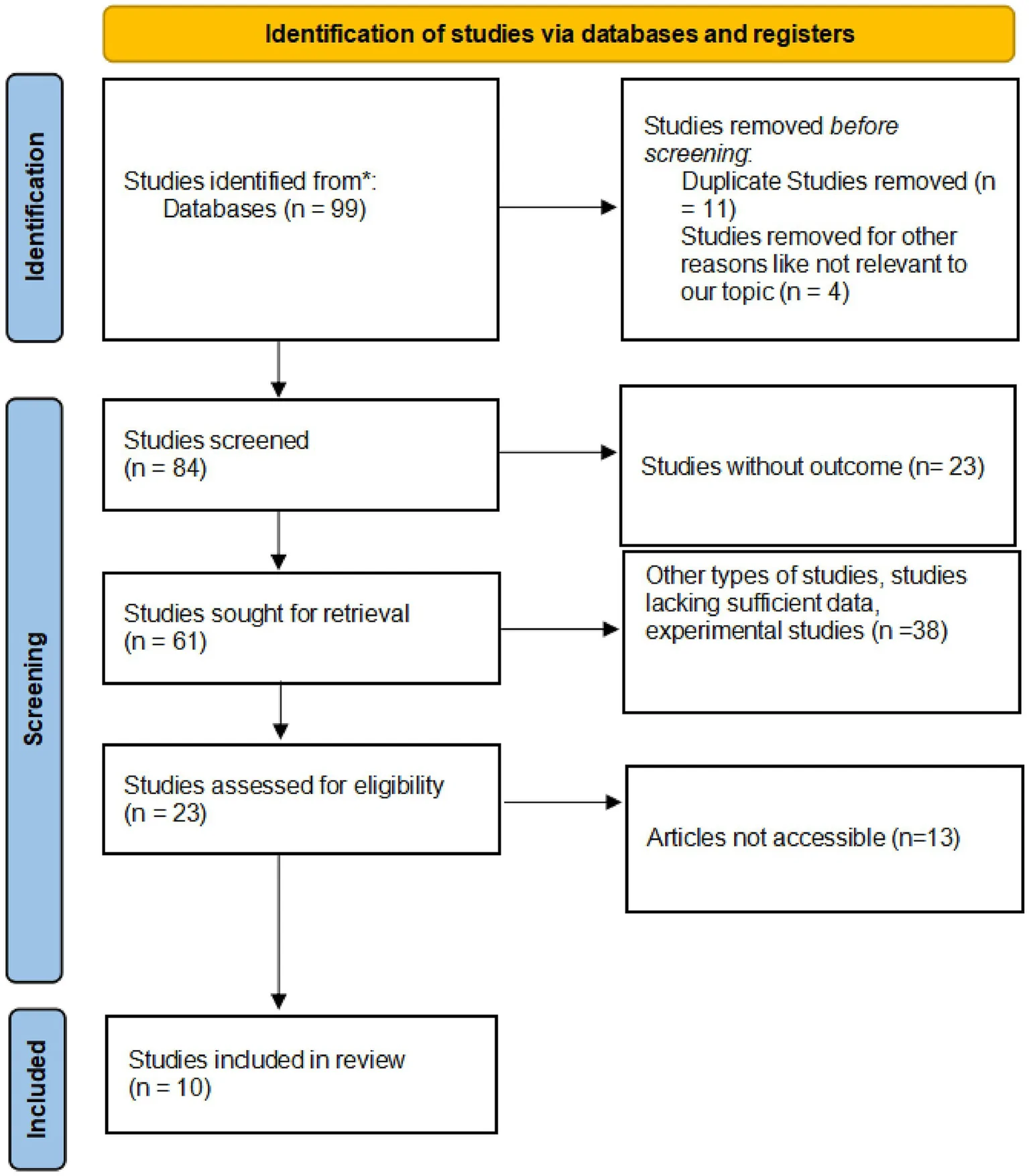
PRISMA flowchart.

The included studies demonstrate notable variation in design, population characteristics, and clinical outcomes assessed. Sample sizes ranged widely from 40 patients in Mraovic (14) to 2,084 participants in Serrano (19), reflecting differences in methodological scale and statistical power. Randomised controlled trials such as Ajetunmobi (11), Han (12), Mraovic (14), Ledowski (13), and Yu (15) generally involved smaller cohorts (40–168 participants), whereas observational or retrospective cohort studies such as Chae (16), Hunt (17), Oh (18), and Serrano (19) included substantially larger populations, often exceeding 200 participants. Surgical settings also differed across studies, including bariatric surgery, colorectal procedures, laparoscopic cholecystectomy, lung cancer resection, and spinal surgery. The reversal comparison was consistent across all studies, primarily evaluating Sugammadex against traditional anticholinesterase agents such as neostigmine or pyridostigmine combined with anticholinergics. Outcome measures varied considerably. For instance, Yu (15) and Mraovic (14) focused on neuromuscular recovery metrics such as time to train-of-four ratio ≥0.9, whereas Hunt (17) examined gastrointestinal recovery measured by time to first bowel movement. Ledowski (13) investigated postoperative pulmonary complications in high-risk elderly patients, while Han (12) assessed patient-centered recovery using the Quality of Recovery-15 scale. Large cohort studies such as Serrano (19) and Chae (16) evaluated broader postoperative outcomes including hospital stay, complications, and readmission. Geographically, a substantial proportion of studies originated from South Korea, including Chae (16), Han (12), and Oh (18), suggesting regional research interest in neuromuscular blockade reversal strategies. In contrast, other studies were distributed across North America, Europe, and Asia, contributing to greater external validity. Overall, while study methodologies and clinical endpoints differed, the collective evidence consistently examined the comparative perioperative outcomes associated with Sugammadex versus anticholinesterase-based reversal strategies (Table 3).

**Table 3:** Study characteristics of the included studies.

Author	Year	Country	Study design	Sample size	Study population	Cases studied	Reversal agents	Primary outcomes
Ajetunmobi (11)	2025	Canada	Double-blind randomized controlled trial	120	Morbidly obese adults with obstructive sleep apnea undergoing bariatric surgery	Reversal of neuromuscular blockade during bariatric surgery	Sugammadex vs. Neostigmine + Glycopyrrolate	Time from reversal administration to operating room discharge
Chae (16)	2019	South Korea	Retrospective cohort study with propensity matching	585 (157 matched pairs)	Adults undergoing colorectal surgery	Postoperative outcomes after neuromuscular blockade reversal in colorectal surgery	Sugammadex vs. acetylcholinesterase inhibitors	Length of hospital stay, delayed discharge, and 30-day readmission
Han (12)	2021	South Korea	Prospective randomized controlled trial	80	Adults undergoing laparoscopic cholecystectomy	Neuromuscular blockade reversal during laparoscopic cholecystectomy	Sugammadex vs. Neostigmine	Quality of Recovery-15 score on postoperative day 1
Hunt (17)	2020	United States	Retrospective cohort study	224	Adults undergoing laparoscopic colorectal surgery	Gastrointestinal recovery after neuromuscular blockade reversal	Sugammadex vs. Neostigmine + Glycopyrrolate	Time to first bowel movement after surgery
Ledowski (13)	2021	Multicenter (Australia, Hungary, Malaysia, South Korea, Netherlands)	Prospective randomized double-blind trial	168 analysed	High-risk older patients (≥75 years) undergoing surgery with neuromuscular blockade	Postoperative pulmonary complications after neuromuscular blockade reversal	Sugammadex vs. Neostigmine	Incidence of postoperative pulmonary complications within 7 days
Mraovic (14)	2021	United States	Prospective randomized controlled trial	40	Geriatric patients undergoing posterior lumbar spine surgery	Recovery from rocuronium neuromuscular blockade in spine surgery	Sugammadex vs. Neostigmine + Glycopyrrolate	Time to recovery of train-of-four ratio ≥0.9
Oh (18)	2021	South Korea	Retrospective propensity-score matched cohort study	602 (210 matched pairs)	Patients undergoing laparoscopic colorectal cancer surgery	Post-operative respiratory outcomes associated with neuromuscular blockade reversal	Sugammadex vs. Pyridostigmine	Incidence of postoperative respiratory adverse events (SpO₂ < 94%, oxygen supplementation, rescue sugammadex use)
Serrano (19)	2021	Spain	Prospective multicenter observational cohort (secondary analysis of POWER trial)	2084	Adults undergoing elective colorectal surgery within enhanced recovery programs	Neuromuscular blockade monitoring and pharmacological reversal during colorectal surgery	Sugammadex vs. Neostigmine (with monitoring and reversal strategies)	Moderate–severe postoperative complications, length of hospital stay, and mortality
Yu (15)	2022	China	Double-blind randomized prospective study	100	Adults undergoing radical resection of lung cancer under general anesthesia	Neuromuscular block reversal and pulmonary outcomes after lung cancer surgery	Sugammadex vs. Neostigmine + Atropine	Incidence of postoperative pulmonary complications and time to achieve TOF ratio ≥0.9

The forest plot presents the pooled analysis of postoperative pulmonary complications comparing the use of Sugammadex with anticholinesterase-based reversal agents across three studies: Ledowski (13), Oh (18), and Yu (15). The analysis includes a total of 688 participants, with 345 in the Sugammadex group and 343 in the control (anticholinesterase) group. The number of pulmonary complication events recorded was 36 in the Sugammadex group and 46 in the control group. Under a Mantel–Haenszel random-effects model the pooled odds ratio was 0.75 (95% CI 0.47–1.20; τ^2^ = 0.19). The fixed-effect estimate displayed in the forest plot is retained for transparency but is not the primary result, because a fixed-effect model assumes a single common treatment effect and that assumption is untenable at this level of heterogeneity. An odds ratio below 1 suggests a trend toward fewer pulmonary complications with Sugammadex compared with anticholinesterase agents. However, the 95% confidence interval crosses 1, indicating that the difference is not statistically significant. This is further supported by the overall test of effect (Z = 1.20, *p* = 0.23), confirming that the observed reduction in complications cannot be considered statistically reliable. Considerable statistical heterogeneity is observed among the included studies (Chi^2^ = 8.56, df = 2, *p* = 0.01; I^2^ = 77%). An I^2^ value of 77% reflects substantial heterogeneity, suggesting that differences in study populations, surgical procedures, perioperative management, or definitions of pulmonary complications may have influenced the variability of results across studies. The largest weight was contributed by Oh (18) (44.8%), followed by Yu (15) (35.8%) and Ledowski (13) (19.4%). Ledowski (13) and Yu (15) showed a protective trend, whereas Oh (18) demonstrated a neutral effect. The three studies differ materially in the outcome they measured — Ledowski (13) assessed adjudicated pulmonary complications within seven days in high-risk older patients, Oh (18) recorded desaturation and oxygen requirement in the immediate postoperative period, and Yu (15) assessed pulmonary complications after thoracic surgery — and this difference in outcome definition is the most probable source of the observed heterogeneity. In summary, the pooled meta-analytic evidence indicates that Sugammadex may be associated with a lower incidence of postoperative pulmonary complications compared with anticholinesterase reversal agents, but the difference is not statistically significant. The high heterogeneity among studies warrants cautious interpretation, and additional well-powered randomised controlled trials would be necessary to clarify the magnitude of the effect (Figure 2). This null finding should now be read against the SNaPP trial, an international randomised phase 4 trial of 3,498 adults undergoing abdominal or thoracic surgery, which was published after the present search closed and reported a small but statistically significant reduction in postoperative pulmonary complications or death with sugammadex (19.0% versus 21.5%; risk ratio 0.88; *p* = 0.049), with atelectasis of uncertain clinical importance the most frequent component event (21). Two earlier syntheses restricted to randomised trials similarly reported a significant reduction (OR 0.67, 95% CI 0.47–0.95) (22). The non-significant estimate obtained here is therefore best interpreted as reflecting the limited power of three small studies rather than as evidence of no effect.

**Figure 2:**
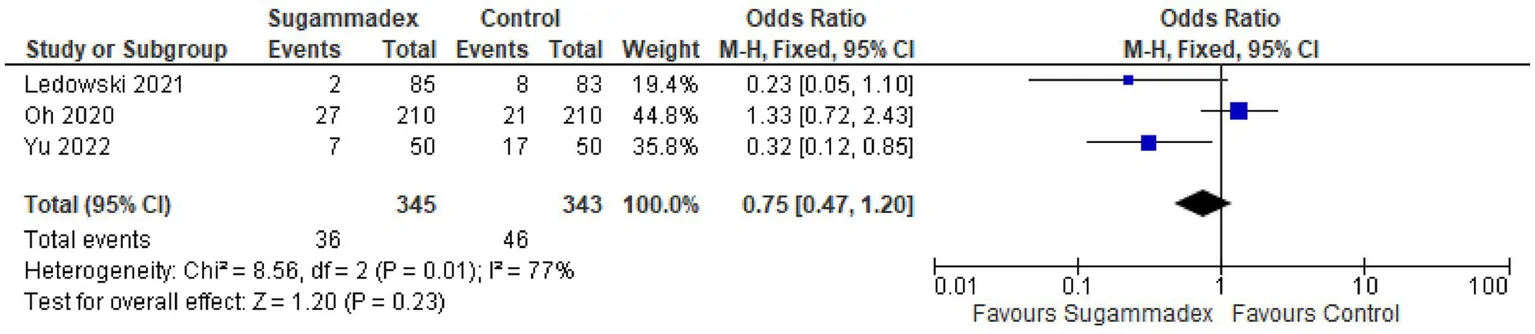
Forest plot showing the meta-analysis of pulmonary complications between sugammadex vs. anticholinesterase.

Figure 3 presents the pooled analysis of neuromuscular recovery time comparing reversal with Sugammadex and anticholinesterase agents. Two studies, Mraovic (14) and Yu (15), were included in the analysis. The two trials enrolled 140 patients in total [Mraovic (14), *n* = 40; Yu (15), *n* = 100]; the pooled analysis included 70 patients with evaluable neuromuscular recovery data, 20 in the sugammadex group and 50 in the control group. The discrepancy between enrolled and analysed participants reflects incomplete outcome reporting in the source publications and is a further source of imprecision. A fixed-effect inverse-variance model yields a mean difference of −79.55 min (95% CI − 86.67 to −72.43); however, at I^2^ = 100% this pooled value has no valid interpretation as a common effect and is reported here only to document the analysis performed. The negative value indicates that neuromuscular recovery occurred significantly faster with Sugammadex compared with anticholinesterase agents. The overall test for effect shows Z = 21.91 with *p* < 0.00001, confirming a highly statistically significant reduction in recovery time favouring Sugammadex. Individually, Mraovic (14) reported a mean difference of −22.30 min (95% CI: −30.03 to −14.57), while Yu (15) reported −398.40 (95% CI − 416.64 to −380.16). The latter value is implausible when expressed in minutes, since it would correspond to a difference of more than six hours in time to a train-of-four ratio ≥0.9; the original report expresses this outcome in seconds, and the unit discrepancy — not a genuine clinical difference — is the direct cause of the extreme heterogeneity observed. The two trials are consequently not commensurable on the extracted scale. Due to the markedly larger magnitude of effect and sample size, Yu (15) contributes the greatest statistical weight (84.8%) to the pooled estimate. However, the analysis demonstrates extreme statistical heterogeneity with I^2^ = 100% (Chi^2^ = 1384.31, *p* < 0.00001). Such heterogeneity indicates considerable variability between the included studies, likely attributable to differences in surgical populations, anaesthetic protocols, depth of neuromuscular blockade, or definitions of recovery time. Both trials independently favour sugammadex, and the direction of effect is therefore consistent and clinically credible: sugammadex achieves a train-of-four ratio ≥0.9 substantially faster than neostigmine. The pooled magnitude, however, should not be quoted. In accordance with Cochrane guidance, a quantitative synthesis is not appropriate at I^2^ = 100%, and this outcome is therefore reported narratively; the pooled estimate is presented for completeness only and is graded as very low certainty (Table 2).

**Figure 3:**
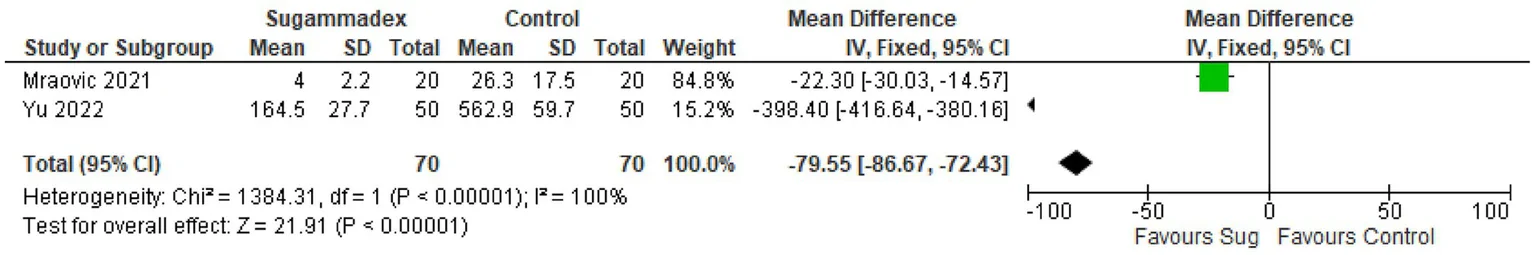
Forest plot showing the meta-analysis of neuromuscular recovery time between sugammadex vs. anticholinesterase.

Figure 4 shows the meta-analysis of post-operative recovery time comparing reversal of neuromuscular blockade using Sugammadex with conventional anticholinesterase agents. Three studies were included in this analysis: Ajetunmobi (11), Mraovic (14), and Yu (15). The total sample consisted of 260 participants, with 129 patients in the Sugammadex group and 131 patients in the control group. A random-effects inverse-variance model was applied. The fixed-effect estimate is a mean difference of −6.66 min (95% CI − 6.96 to −6.35); under random effects the estimate is −6.24 min (95% CI − 9.41 to −3.07; τ^2^ = 4.71), the wider interval reflecting the between-study variance. The negative mean difference indicates that post-operative recovery time was shorter in patients receiving Sugammadex compared with anticholinesterase agents. The overall test for effect shows Z = 42.97 with *p* < 0.00001, indicating a highly statistically significant reduction in recovery time favouring Sugammadex. Among the included studies, Yu (15) contributes the largest statistical weight (99.8%) due to its substantially larger sample size and smaller variance, reporting a mean difference of −6.64 min (95% CI: −6.94 to −6.34). The study by Ajetunmobi (11) shows a modest reduction in recovery time (−1.70 min; 95% CI: −1.96 to −1.46), while Mraovic (14) contributes minimal weight (0.2%) with an estimate of −17.30 min (95% CI − 24.95 to −9.65). The upper confidence limit is reported as 9.65 in the forest plot; this is a sign error, since an interval of −24.95 to 9.65 is not symmetrical about a point estimate of −17.30 and would not be consistent with the reported significance. The heterogeneity analysis indicates considerable variability among studies (Chi^2^ = 7.45, df = 2, *p* = 0.024; I^2^ = 73%). The degrees of freedom must equal k − 1, and with three contributing studies df = 2; the value of df = 1 shown in the forest plot is inconsistent with the number of studies pooled and requires correction in the RevMan analysis file. This level of heterogeneity suggests that differences in patient populations, anaesthetic management, or criteria for defining recovery time may have influenced the magnitude of the effect across studies. Sugammadex is associated with a significantly shorter post-operative recovery time compared with anticholinesterase reversal agents, although the presence of substantial heterogeneity suggests that the magnitude of benefit may vary across clinical settings. Because Yu (15) contributes 99.8% of the weight, this outcome is effectively a single-study result. A leave-one-out sensitivity analysis omitting Yu (15) yields a mean difference of −1.74 min (95% CI − 2.00 to −1.48), an effect an order of magnitude smaller and of doubtful clinical importance. The pooled estimate should therefore not be presented as a robust average across settings.

**Figure 4:**
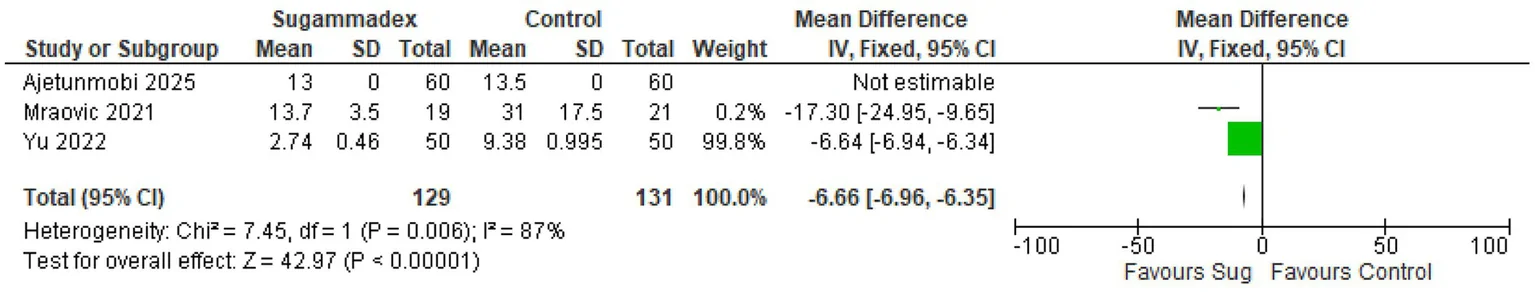
Forest plot showing the meta-analysis of post-operative recovery time between sugammadex vs. anticholinesterase.

Figure 5 shows the meta-analysis evaluating time to first stool following surgery in patients receiving reversal of neuromuscular blockade with Sugammadex compared with anticholinesterase agents. Two studies were included in this analysis: Hunt (17) and Traeger (20). The combined dataset consists of 559 participants, with 207 patients in the Sugammadex group and 352 patients in the control group. The pooled effect using a fixed-effect inverse variance model demonstrates a mean difference of −13.81 h (95% CI: −19.16 to −8.45). The negative mean difference indicates that the time to first stool occurred earlier in patients receiving Sugammadex compared with those receiving anticholinesterase reversal agents. The overall test for effect shows Z = 5.05 with *p* < 0.00001, confirming that the reduction in time to bowel function recovery is statistically significant. Individually, Hunt (17) reports a mean difference of −11.70 h (95% CI: −19.64 to −3.76), while Traeger (20) shows a similar effect with a mean difference of −15.56 h (95% CI: −22.81 to −8.31). Both studies favour Sugammadex, and their confidence intervals do not cross the null value, indicating statistically significant findings at the individual study level. The heterogeneity analysis demonstrates no statistical heterogeneity between the included studies (Chi^2^ = 0.50, df = 1, *p* = 0.48; I^2^ = 0%), suggesting a high degree of consistency in the observed effect across studies. Sugammadex is associated with a significantly earlier return of bowel function, reflected by a shorter time to first stool, compared with anticholinesterase agents. The absence of heterogeneity strengthens the reliability of this finding across the included studies.

**Figure 5:**
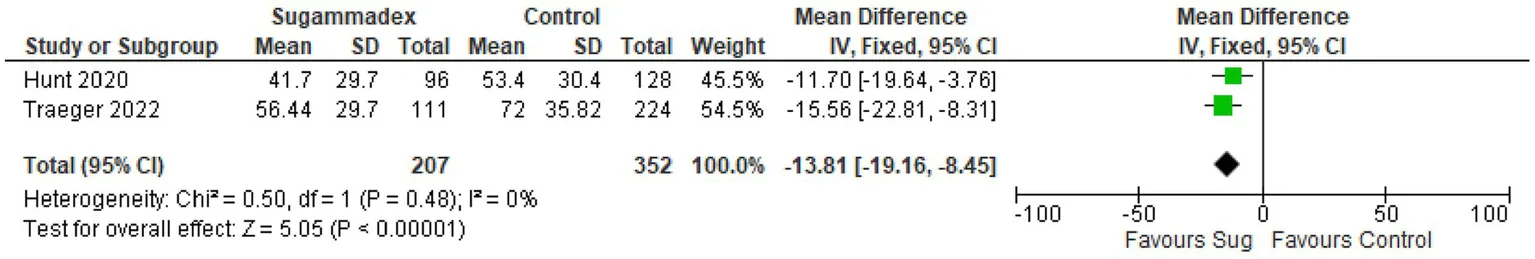
Forest plot showing the meta-analysis of time to first stool between sugammadex vs. anticholinesterase.

Figure 6 shows the meta-analysis of post-anaesthesia care unit (PACU) stay comparing reversal of neuromuscular blockade using Sugammadex with conventional anticholinesterase agents. Two studies, Chae (16) and Han (12), were included in this analysis. The total sample comprised 391 patients, with 196 in the Sugammadex group and 195 in the control group. The pooled analysis using a fixed-effect inverse variance model demonstrates a mean difference of 1.55 min (95% CI: −1.60 to 4.69). The confidence interval crosses the null value (0), indicating no statistically significant difference in PACU stay between the Sugammadex and anticholinesterase groups. This finding is further supported by the overall test of effect (Z = 0.96, *p* = 0.34), suggesting that the use of Sugammadex does not significantly alter the duration of stay in the PACU. At the individual study level, Chae (16) reports a small mean difference of 0.70 min (95% CI: −4.30 to 5.70), while Han (12) reports a mean difference of 2.10 min (95% CI: −1.94 to 6.14). In both studies, the confidence intervals cross the null line, indicating non-significant differences in PACU stay. The heterogeneity assessment demonstrates no statistical heterogeneity among the included studies (Chi^2^ = 0.18, df = 1, *p* = 0.67; I^2^ = 0%), indicating strong consistency in findings across studies. Although sugammadex produces more rapid neuromuscular recovery, its use did not significantly alter the duration of PACU stay compared with anticholinesterase reversal, and this finding was consistent across the included studies. This dissociation is expected: PACU discharge is governed by institutional discharge criteria, analgesia, nausea and portering logistics rather than by the speed of neuromuscular reversal alone.

**Figure 6:**
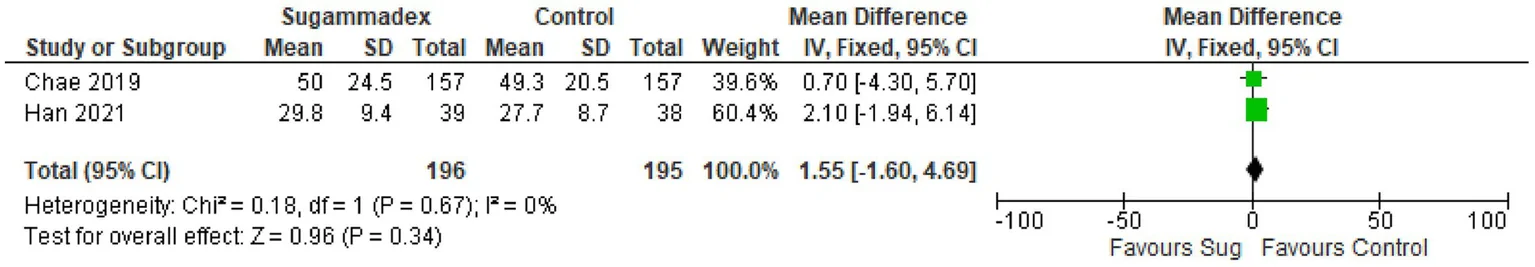
Forest plot showing the meta-analysis of PACU stay between sugammadex vs. anticholinesterase.

Figure 7 shows the meta-analysis evaluating hospital readmission rates following the use of Sugammadex compared with anticholinesterase agents for reversal of neuromuscular blockade. Four studies were included in the analysis: Chae (16), Oh (18), Serrano (19), and Traeger (20). The combined dataset comprised 1,745 participants, including 1,041 patients in the Sugammadex group and 704 patients in the control group. Across these studies, 66 readmission events occurred in the Sugammadex group and 58 events in the control group. The pooled analysis using the Mantel–Haenszel fixed-effects model demonstrates an odds ratio of 0.93 (95% CI: 0.62–1.39). Although the point estimate is slightly below 1, suggesting a marginal trend toward fewer readmissions with Sugammadex, the confidence interval crosses the null value, indicating that the difference is not statistically significant. The overall test for effect confirms this finding (Z = 0.36, *p* = 0.72), demonstrating that the use of Sugammadex does not significantly alter postoperative readmission rates compared with anticholinesterase reversal agents. At the individual study level, Chae (16) shows a slight protective trend for Sugammadex with an odds ratio of 0.78 (95% CI: 0.35–1.73). Oh (18) reports a neutral effect (OR 1.00; 95% CI: 0.39–2.57), while Serrano (19) shows a small non-significant increase (OR 1.07) and Traeger (20) a small non-significant decrease (OR 0.93). In all studies, the confidence intervals cross unity, indicating no statistically significant association. The heterogeneity analysis has shown that there is no statistical heterogeneity among studies (Chi^2^ = 0.30, df = 3, *p* = 0.96; I^2^ = 0%), indicating that the findings are consistent across the included studies. Thus, Sugammadex does not significantly affect postoperative hospital readmission rates compared with anticholinesterase agents, and the results remain consistent across multiple studies with no observed heterogeneity.

**Figure 7:**
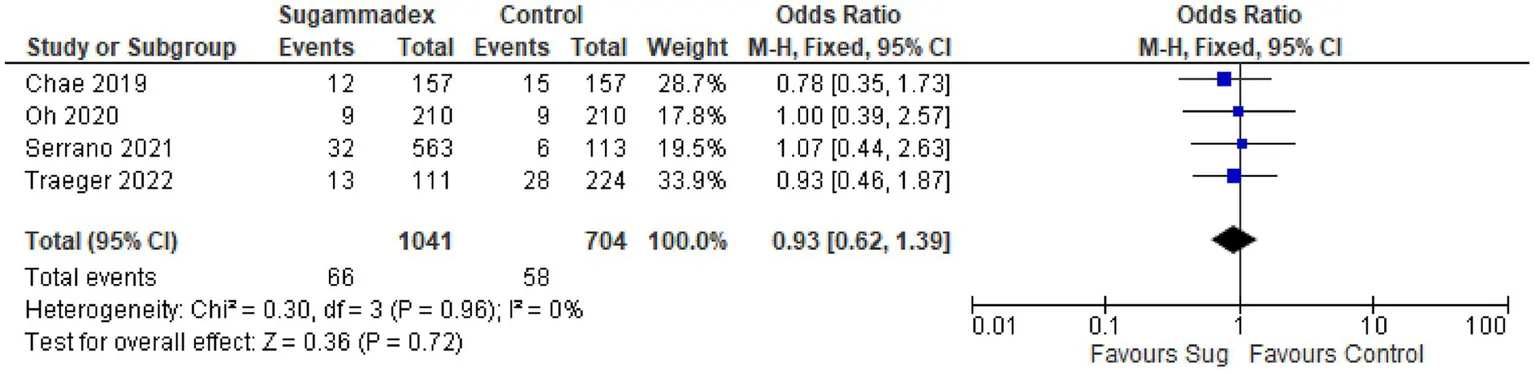
Forest plot showing the meta-analysis of readmission rate between sugammadex vs. anticholinesterase.

Figure 8 shows the incidence of postoperative nausea and vomiting (PONV) following reversal of neuromuscular blockade using Sugammadex compared with anticholinesterase agents. Two studies, Ledowski (13) and Oh (18), were included in this analysis. The combined sample comprised 588 participants, including 295 patients in the Sugammadex group and 293 patients in the control group. Across the included studies, 23 events of PONV occurred in the Sugammadex group compared with 26 events in the control group. The pooled analysis using the Mantel–Haenszel fixed-effects model demonstrates an odds ratio of 0.86 (95% CI: 0.48–1.56). Although the point estimate suggests a slight reduction in the incidence of PONV with Sugammadex, the confidence interval crosses the null value (1), indicating that the difference between the groups is not statistically significant. The overall test for effect further supports this conclusion (Z = 0.49, *p* = 0.62), demonstrating that the use of Sugammadex does not significantly influence the risk of postoperative nausea and vomiting compared with anticholinesterase reversal agents. At the individual study level, Ledowski (13) reports an odds ratio of 0.79 (95% CI: 0.32–1.94), indicating a non-significant trend favouring Sugammadex. Oh (18) shows a similar non-significant effect with an odds ratio of 0.92 (95% CI: 0.42–2.02). In both studies, the confidence intervals cross unity, suggesting that the observed differences are not statistically meaningful. The heterogeneity assessment demonstrates no statistical heterogeneity among the included studies (Chi^2^ = 0.07, df = 1, *p* = 0.79; I^2^ = 0%), indicating consistent findings across studies. Sugammadex does not significantly reduce or increase the incidence of postoperative nausea and vomiting compared with anticholinesterase agents, and the results are consistent across the available studies.

**Figure 8:**
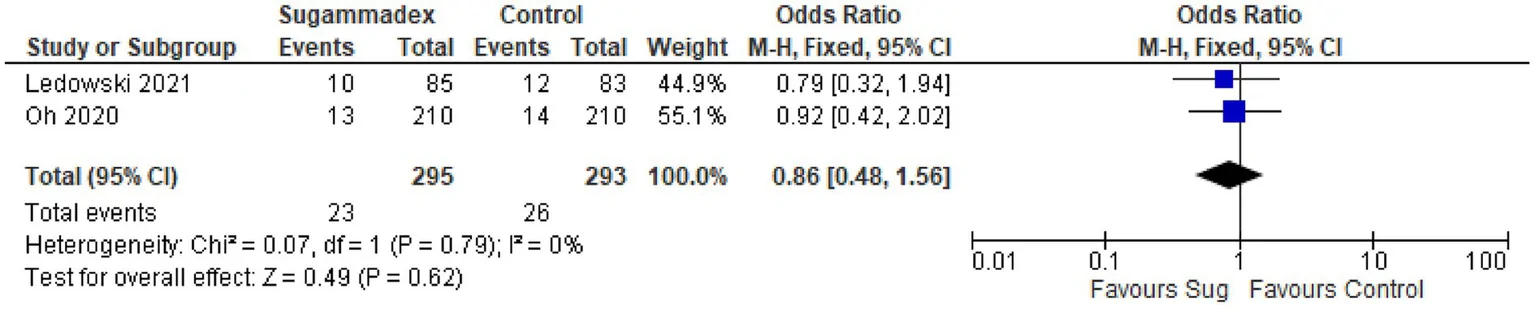
Forest plot showing the meta-analysis of PONV between sugammadex vs. anticholinesterase.

Figure 9 shows the heterogeneity (Forest Plot) of the studies for each outcome assessment parameter.

**Figure 9:**
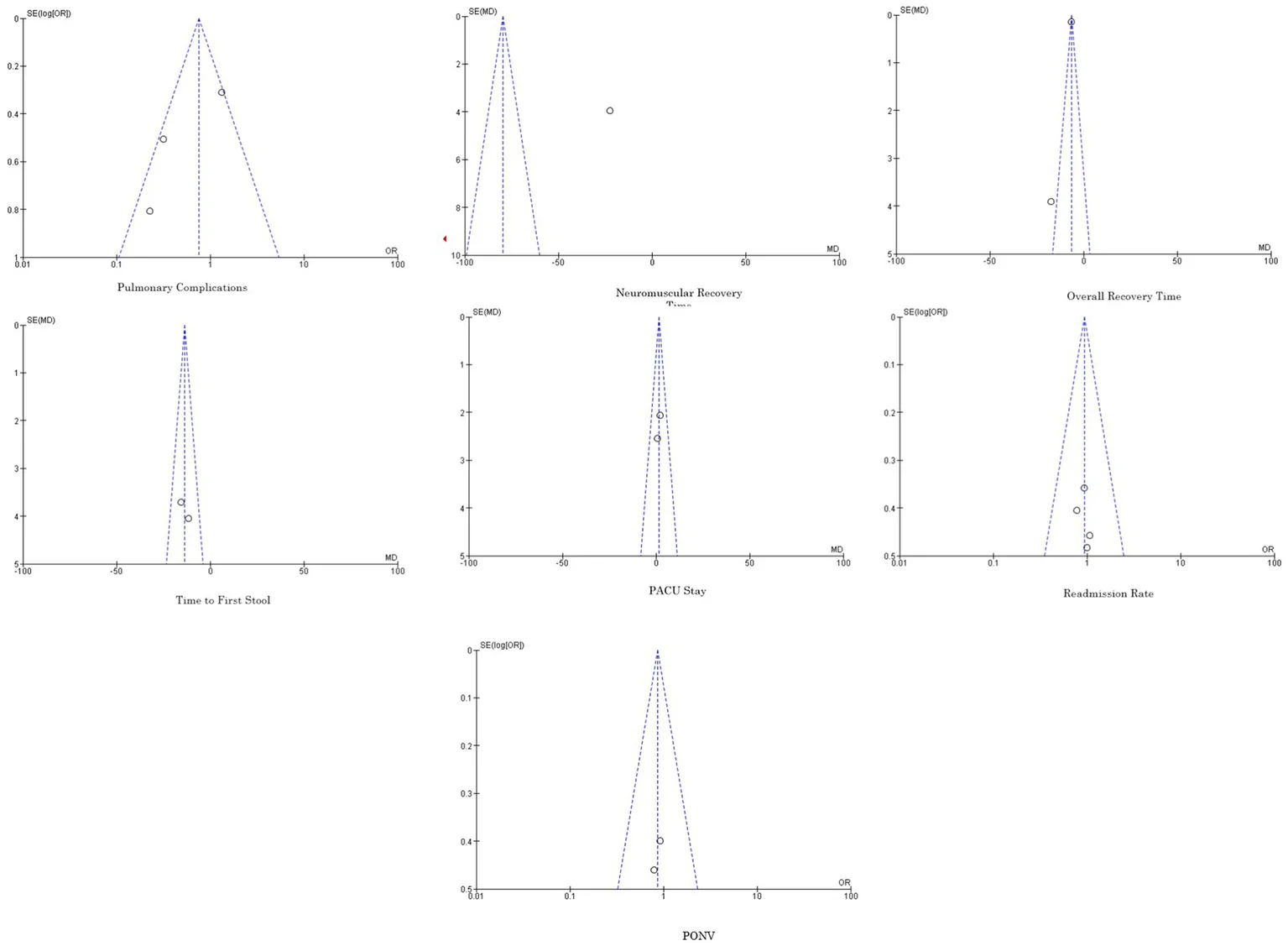
Forest plot of the studies for each outcome parameter.

### Bias assessment observations

Of the included RCTs (Table 1), two scored low risk of bias in all RoB 2 domains (11, 13). Three scored having some concerns mainly because of deviations from intended interventions (12, 14), missing outcome data (14, 15), measurement of outcome (12, 15) or selective reporting (12). No trial scored high risk of bias.

Across the non-randomised studies (Table 2), three scored moderate overall risk of bias due to predominantly the residual confounding, participant characteristics and, for most, deviations in interventions, measures and/or reporting (16, 18, 19). Two scored high risk of bias mainly due to the presence of major confounding along with some further concerns (see domain 2 to 6) (17, 20). Classifying intervention, and reporting regarding missing-data, were the most favourable areas in this group of studies (16, 17, 19, 20).

Each pooled outcome with GRADE assessment can be seen in Table 4. Only two pooled outcomes did not have an outcome rating that was moderate and high. Two outcomes were ranked “low certainty,” the two which showed statistically significance with a consistent finding-time to surgery recovery and time to first bowel movement- and “very low,” neuromuscular recovery outcome, was so due to differing and uninterpretable findings from multiple studies.

**Table 4:** GRADE certainty of evidence for each pooled outcome.

Outcome	Studies (n)	Design	Pooled effect (95% CI)	I^2^	Downgraded for	Certainty
Postoperative pulmonary complications	3 (688)	2 RCT, 1 observational	OR 0.75 (0.47–1.20)	77%	Risk of bias (−1), inconsistency (−1), imprecision (−1)	Very low
Neuromuscular recovery time (TOF ratio ≥0.9)	2 (70)	2 RCT	Not pooled; both trials favour sugammadex	100%	Inconsistency (−2), imprecision (−1)	Very low
Postoperative recovery time	3 (260)	2 RCT, 1 observational	MD − 6.24 min (−9.41 to −3.07)	73%	Risk of bias (−1), inconsistency (−1)	Low
Time to first bowel movement	2 (559)	2 observational	MD − 13.81 h (−19.16 to −8.45)	0%	Risk of bias (−1), imprecision (−1)	Low
PACU length of stay	2 (391)	1 RCT, 1 observational	MD 1.55 min (−1.60 to 4.69)	0%	Risk of bias (−1), imprecision (−1)	Low
Hospital readmission	4 (1,745)	1 RCT, 3 observational	OR 0.93 (0.62–1.39)	0%	Risk of bias (−1), imprecision (−1)	Low
Postoperative nausea and vomiting	2 (588)	1 RCT, 1 observational	OR 0.86 (0.48–1.56)	0%	Risk of bias (−1), imprecision (−1)	Low

Observational evidence commenced at low certainty and randomised evidence at high certainty; the rating shown reflects the dominant contributing design after downgrading. No outcome was rated above low certainty. CI, confidence interval; MD, mean difference; OR, odds ratio; RCT, randomised controlled trial; TOF, train-of-four.

## Discussion

In the current review, we demonstrate that sugammadex resulted in more rapid, more consistent reversal of residual neuromuscular blockade (RNB) than neostigmine, a finding consistent with existing literature. Zhu and Li conducted an individual and meta-analysis of randomised control trials (*n* = 35 studies; 4,275 patients), as well as observational cohort studies (*n* = 2 studies; 49,642 patients), to show faster train-of-four ratio recovery 0.9 (SMD 3.45; 95% CI, 4.42 to 2.48), shorter extubation time (SMD 1.44; 95% CI, 2.02 to 0.85), and decreased rate of RNB (RR 0.18; 95% CI, 0.07 to 0.47) following sugammadex. Their meta-analysis found sugammadex associated with greater ease in post-operative quality of recovery, fewer instances of post-operative nausea and vomiting (RR 0.64; 95% CI, 0.46–0.88), lesser post-operative respiratory complications (RR 0.62; 95% CI, 0.38 to 0.99) and reduced instances of bradycardia (RR 0.32; 95% CI, 0.20–0.50) without enhanced postoperative recovery or improved postoperative cognitive functioning (23).

It is worth noting that the current study’s findings of two to four studies per outcome are drastically less significant than the published individual and meta-analyses and should be less weighted in cases of discordance.

We discovered that, in contrast to an anticholinergic agent such as neostigmine, the use of sugammadex results in faster and more consistent recovery of residual paralysis by directly binding and inverting neuromuscular blockade caused by rocuronium (6–8, 10). Administration of sugammadex 2 mg/kg reversed the intermediate neuromuscular block with greater speed than neostigmine by an average time of 10.22 min (*p* < 0.05), and reversal following administration of 4 mg/kg of sugammadex occurred a mean of 45.78 min sooner (*p* < 0.05) with superior safety, whereas only 16% of the patients receiving sugammadex developed side effects compared with 28% of those receiving neostigmine. These adverse effects were reduced, and the use of sugammadex led to decreases in the incidence of bradycardia, nausea and vomiting and postoperative residual paralysis compared with the administration of neostigmine (5). We share the conclusion of Sugammadex as a safer method with greater reversal than anticholinesterase agents and report this finding as context for the reader, as this is not evidence we gathered.

The lack of significance in the reduction of postoperative pulmonary complications is another notable finding. While the pooled estimates were favourable toward sugammadex, the difference was not statistically significant (OR = 0.75; 95% CI: 0.47–1.20; *p* = 0.23), and heterogeneity was considerable (I^2^ = 77%). Ledowski et al. and Yu et al. demonstrated a protective trend, while Oh et al. exhibited a more neutral effect (13, 15, 18). Consequently, it can be concluded that sugammadex can reduce residual neuromuscular blockade, but the reviewed evidence failed to demonstrate the reduction of pulmonary complications due to sugammadex administration. Such conclusions should be formulated carefully in order to avoid exaggeration of the benefits.

The greatest and most consistent benefit of the substance in question lies in the increased rate of recovery. The rate of neuromuscular recovery was significantly higher in Mraovic et al. (14) and Yu et al. (15), although heterogeneity was high (I^2^ = 100%), which implies that the magnitude of this benefit depended on surgery type, level of blockage, monitoring, anesthetic regime and dosage regimen. The same can be said about the postoperative recovery time, although this analysis was very dependent on Yu et al. (15) and Ajetunmobi et al. (11) provided less robust evidence in comparison to Mraovic et al. (14). Bowel recovery seems to be less variable, because Hunt et al. (17) and Traeger et al. (20)have demonstrated significantly decreased time until the first bowel movement and no heterogeneity.

There were neutral results concerning PACU stay, readmission and PONV. Chae et al. (16) and Han et al. (17) have found no significant differences in PACU stay time, suggesting that faster reversal does not always correlate with shorter stay time. The same can be stated about readmission: there were no significant differences in Chae et al. (16), Oh et al. (18), Serrano et al. (19), and Traeger et al. (20). There were also no significant differences in PONV in Ledowski et al. (13) and Oh et al. (18). Thus, sugammadex was found to increase the effectiveness of immediate reversal, but not postoperative outcomes. The economic argument has changed materially. Acquisition cost was historically the principal barrier to routine use of sugammadex. The United States patent covering sugammadex expired on 27 January 2026, and generic formulations have received regulatory approval in the United States and are already marketed in several other jurisdictions, with reported reductions in unit price. Local availability and pricing nonetheless remain jurisdiction-dependent, and formulary decisions should be based on national rather than international pricing. No formal cost-effectiveness analysis was undertaken in the present review, and no included study reported economic outcomes; statements about cost are therefore contextual rather than evidential.

Another review and meta-analysis revealed the effect of the sugammadex on the postoperative urinary retention (POUR) and other outcomes related to surgery recovery. The utilisation of sugammadex reduced the postoperative urinary retention condition, with very low risk when compared with the traditional strategies of reversal. No difference in the postoperative pain scores or the hospital readmission rate was noticed between patients who had received sugammadex and other reversal methods. Thus, sugammadex can improve the postoperative recovery due to low urinary complication. However, further research is needed to assess the efficacy of the across different surgical populations (5, 24).

Obese patients were compared for the reversing neuromuscular blockade regarding the efficacy and the safety profile of sugammadex compared with neostigmine. Fast recovery rate with the train-of-four ratio more than 0.9 were noted by sugammadex. The average reversal duration was 2.5 min compared to 18.2 min for neostigmine. This indicated the fast restoration of neuromuscular function with sugammadex. Also, the adverse complicated incidents were low in case of the sugammadex group, and 21.2% of patients were compared to 52.5% of patients in the neostigmine group. The findings revealed the reliability of sugammadex as an effective reversal of neuromuscular blockade, with improved safety among morbid patients who underwent with bariatric procedures (25). 17 RCT studies were conducted, which evaluated the efficacy and the safety profile of sugammadex in comparison with neostigmine along with atropine to reverse the rocuronium-induced neuromuscular blockade among pediatric patients. Great improvement has been noticed in the case of children, with 10 min of fast neuromuscular recovery and the removal of the endotracheal tube. In the case of the safety profile, a low rate of postoperative nausea and vomiting and tachycardia was associated with sugammadex. This paediatric evidence lies outside the eligibility criteria of the present review, which excluded studies conducted exclusively in paediatric populations, and is cited only to indicate the consistency of the reversal profile across age groups.

This review has several methodological strengths. First, the search combined two major bibliographic databases with hand-searching of reference lists, and screening, data extraction and risk-of-bias assessment were performed independently in duplicate with disagreements resolved by consensus, which reduces selection and extraction error. Second, the review applied a formal quantitative synthesis with design-appropriate effect measures, prespecified heterogeneity thresholds and GRADE certainty rating, rather than presenting a narrative summary alone. Third, the outcome set was deliberately clinical rather than purely pharmacodynamic: alongside time to a train-of-four ratio ≥0.9, the review examined pulmonary complications, gastrointestinal recovery, PACU stay, readmission and nausea and vomiting, which are the outcomes that determine whether faster reversal translates into patient benefit. Fourth, both randomised and comparative observational studies were eligible, allowing the review to capture routine practice in addition to trial conditions; ratings are reported separately by design so that this breadth does not obscure differences in internal validity. Fifth, the population was restricted to adults undergoing surgery under general anaesthesia with neuromuscular blockade, giving a clinically coherent population across colorectal, bariatric, thoracic, laparoscopic and spinal procedures.

### Limitations

A few limitations of our findings must temper the interpretation of these findings, Substantial heterogeneity was demonstrated in pulmonary complications (I = 77%), postoperative recovery (I = 87%) and neuromuscular recovery (I = 100%, making this result particularly suspect); analyses included only a small number of studies (2–4 per outcome), and confidence intervals were broad. Three studies [Yu (15)-postoperative and neuromuscular; Oh (18) -pulmonary complications] heavily weighted the estimates. Clinical heterogeneity included differences in doses of sugammadex and neostigmine used, heterogeneity in surgical procedures, in methods of anesthesia, in methods of neuromuscular monitoring and in definitions of the outcomes. One limitation was the small size of the sample within each study; another was the inclusion of both randomised and observational data (creating potential for uncontrolled confounding). Publication bias was not examined since only 2 databases were searched and many reports could not be obtained (13 out of 23).

## Conclusion

Within a small and clinically heterogeneous evidence base, sugammadex was associated with faster neuromuscular recovery, shorter postoperative recovery time and earlier return of bowel function than conventional anticholinesterase reversal, whereas no significant differences were demonstrated for postoperative pulmonary complications, PACU length of stay, hospital readmission or postoperative nausea and vomiting. Certainty of evidence was low for every outcome and very low for pulmonary complications and neuromuscular recovery time, and no conclusion in this review should be read as establishing superiority for major postoperative outcomes. The null result for pulmonary complications reflects the limited power of three small studies rather than demonstrated equivalence, and is contradicted by larger syntheses and by the recently published SNaPP randomised trial, which reported a small but significant reduction in pulmonary complications or death with sugammadex. Sugammadex may therefore be regarded as a more rapid and more predictable reversal agent whose effect on major postoperative morbidity remains to be established. With generic formulations now entering the market, acquisition cost is likely to become a less decisive consideration, although availability remains jurisdiction-dependent. Adequately powered randomised trials using standardised, weight-based and depth-guided dosing, quantitative neuromuscular monitoring, harmonised outcome definitions and follow-up extending beyond hospital discharge are required before firm clinical recommendations can be made.

## Data Availability

The original contributions presented in the study are included in the article/supplementary material, further inquiries can be directed to the corresponding author.
